# DDBJ update: the Genomic Expression Archive (GEA) for functional genomics data

**DOI:** 10.1093/nar/gky1002

**Published:** 2018-10-24

**Authors:** Yuichi Kodama, Jun Mashima, Takehide Kosuge, Osamu Ogasawara

**Affiliations:** DDBJ Center, National Institute of Genetics, Shizuoka 411-8540, Japan

## Abstract

The Genomic Expression Archive (GEA) for functional genomics data from microarray and high-throughput sequencing experiments has been established at the DNA Data Bank of Japan (DDBJ) Center (https://www.ddbj.nig.ac.jp), which is a member of the International Nucleotide Sequence Database Collaboration (INSDC) with the US National Center for Biotechnology Information and the European Bioinformatics Institute. The DDBJ Center collects nucleotide sequence data and associated biological information from researchers and also services the Japanese Genotype–phenotype Archive (JGA) with the National Bioscience Database Center for collecting human data. To automate the submission process, we have implemented the DDBJ BioSample validator which checks submitted records, auto-corrects their format, and issues error messages and warnings if necessary. The DDBJ Center also operates the NIG supercomputer, prepared for analyzing large-scale genome sequences. We now offer a secure platform specifically to handle personal human genomes. This report describes database activities for INSDC and JGA over the past year, the newly launched GEA, submission, retrieval, and analysis services available in our supercomputer system and their recent developments.

## INTRODUCTION

The DNA Data Bank of Japan (DDBJ, https://www.ddbj.nig.ac.jp) (1) is a public nucleotide sequence database established at the National Institute of Genetics (NIG, https://www.nig.ac.jp). Since 1987, the DDBJ Center has been collecting annotated nucleotide sequences as its traditional database service. This endeavor is conducted in collaboration with GenBank (2) at the National Center for Biotechnology Information (NCBI) and with the European Nucleotide Archive (ENA) (3) at the European Bioinformatics Institute (EBI). This collaborative framework is known as the International Nucleotide Sequence Database Collaboration (INSDC) (4), and its product database is called the International Nucleotide Sequence Database (INSD).

Within the INSDC framework, the DDBJ Center also services the DDBJ Sequence Read Archive (DRA) for raw sequencing data and alignment information from high-throughput sequencing platforms (5), BioProject for sequencing project metadata, and BioSample for sample information (1,6). This comprehensive resource of nucleotide sequences and associated biological information complies with INSDC policy guaranteeing free and unrestricted access to data archives (7).

In July 2018, the DDBJ Center launched a new public database, the Genomic Expression Archive (GEA, https://www.ddbj.nig.ac.jp/gea), which collects functional genomics data from microarray and high-throughput sequencing experiments. Besides the Gene Expression Omnibus (GEO) at the NCBI (8) and ArrayExpress at the EBI (9), the GEA issues accession numbers to functional genomics experiments, whose data are associated with metadata in a structured and standardized MAGE-TAB format (10), and public GEA data will be indexed by ArrayExpress. For publications under review, submitters can allow journal reviewers anonymous access to private GEA data cited in their manuscripts. With the GEA launch, the DDBJ Center now covers the archiving of sequences with functional annotation (traditional database) and molecular abundance (GEA).

In addition to these unrestricted-access databases, the DDBJ Center also services a controlled-access database, the Japanese Genotype–phenotype Archive (JGA, https://www.ddbj.nig.ac.jp/jga), in collaboration with the National Bioscience Database Center (NBDC, https://biosciencedbc.jp/en/) at the Japan Science and Technology Agency (1,11). The JGA stores genotype and phenotype data from human individuals who have signed consent agreements authorizing data usage for specific research only. The NBDC provides guidelines and policies for sharing human-derived data (https://humandbs.biosciencedbc.jp/en/guidelines) and reviews data submission and usage requests.

The DDBJ Center operates a commodity-based computer cluster, the NIG supercomputer. It provides an analytical environment for domestic researchers to examine large-scale biology data from its archival databases with preinstalled bioinformatics tools.

**Figure 1.**
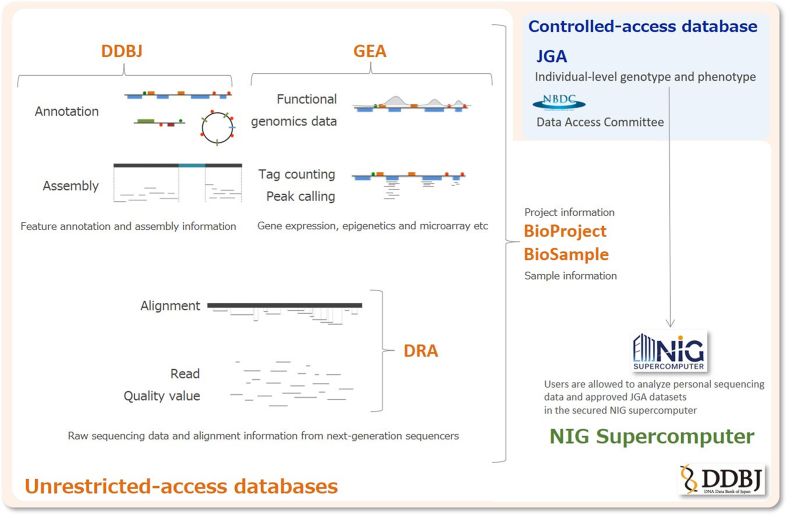
Database and supercomputer services of the DDBJ Center. The unrestricted-access databases (DRA, DDBJ, GEA, BioProject and BioSample) are illustrated with their data scopes. The controlled-access database (JGA) is operated in collaboration with NBDC, whose Data Access Committee reviews data submission and usage requests according to the NBDC guidelines for sharing human-derived data. The NIG supercomputer is a commodity-based cluster designed for analyzing large-scale sequencing data. In the secure platform designed for analyzing personal human genomes, users are allowed to analyze approved JGA datasets.

In the present article, we provide an update of the above services at the DDBJ Center, highlighting the new database resource: the GEA. All resources mentioned in this article are available at https://www.ddbj.nig.ac.jp, and most of the archival data can be downloaded from ftp://ftp.ddbj.nig.ac.jp. Major database and supercomputer services are summarized in Figure 1.

## DDBJ ARCHIVAL DATABASES

### Data contents: unrestricted- and controlled-access databases

From June 2017 to May 2018, the traditional DDBJ accepted 6011 nucleotide data submissions consisting of 5 926 950 entries, most of which were from Japanese research groups (4784 submissions; 79.6%). The DDBJ periodical release includes both conventional sequence and bulk sequence data such as whole-genome shotgun (WGS), transcriptome shotgun assembly (TSA), and targeted locus study (TLS) but does not include third-party data (TPA) records (12). Between June 2017 and May 2018, the DDBJ periodical release increased from 689 916 250 to 1 564 840 159 entries and from 1 333 798 893 388 to 3 795 161 222 944 base pairs. The DDBJ contributed 4.67% of the entries and 3.25% of the total base pairs in the INSD nucleotide sequence data. A detailed statistical breakdown of records is present on the DDBJ website (https://www.ddbj.nig.ac.jp/stats/release-e.html#total_data).

During June 2017 and May 2018, high-throughput sequencing data consisting of 43 174 runs have been registered to the DRA. As of 24 August 2018, the DRA has distributed 3.8 PB of sequencing data in SRA (2.7 PB) and FASTQ (1.1 PB) formats.

The JGA is a controlled-access database for human genotype and phenotype data (11) similar to the database of Genotypes and Phenotypes (dbGaP) at the NCBI (13) and the European Genome-phenome Archive at the EBI (14). As of 24 August 2018, the JGA has archived 146 studies, 248 043 samples, and 96 TB of individual-level human datasets submitted by Japanese researchers. The summaries of 88 studies are publicly available both on the JGA (https://ddbj.nig.ac.jp/jga/viewer/view/studies) and NBDC (https://humandbs.biosciencedbc.jp/en/data-use/all-researches) websites. A large-scale dataset of the BioBank Japan project (15) is available under JGA study accession number JGAS00000000114. This includes the microarray genotyping data of 182 505 individuals, the whole genome sequencing data of 1026 individuals, and 58 quantitative traits associated with 47 diseases of 200 849 individuals. To access the individual-level data of these public studies, users are required to send data usage requests to the NBDC (https://humandbs.biosciencedbc.jp/en/data-use).

### The Genomic Expression Archive

The GEA is a public archive for functional genomics experiments including gene expression, epigenetics, and genome-protein interactions. Along with the GEO and ArrayExpress, the GEA supports two community-derived reporting standards—the Minimum Information About a Microarray Experiment (MIAME) (16) and the Minimum Information about a high-throughput nucleotide SEQuencing Experiment (MINSEQE, http://fged.org/projects/minseqe)—for unambiguous interpretation and data usage. These guidelines specify the provision of critical experimental elements in raw data, processed data, sample annotation, and protocols.

**Figure 2.**
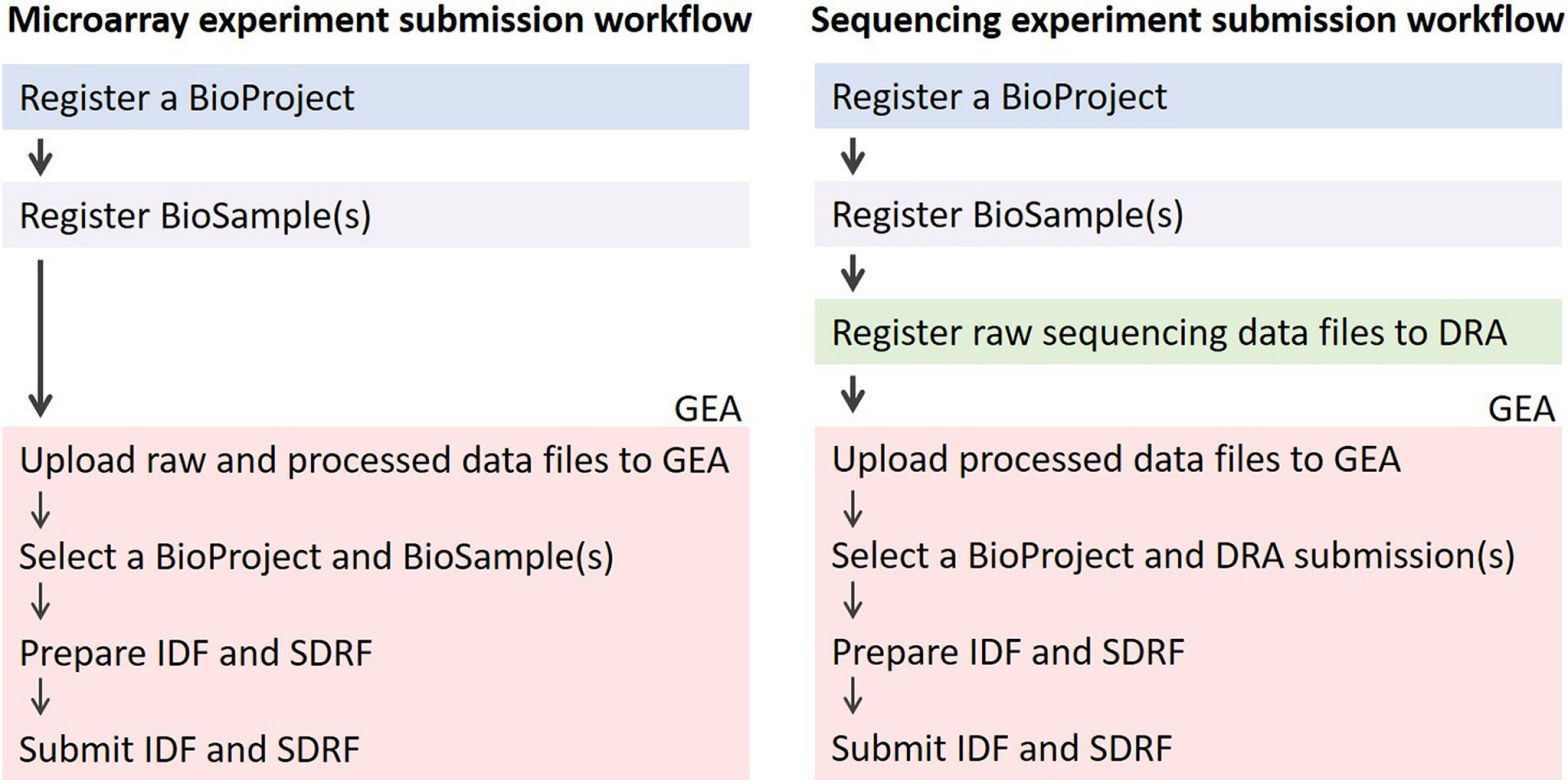
GEA submission workflow of microarray and sequencing experiments. Registration of BioProject and BioSample(s) are mandatory prior to the submission of both microarray and sequencing experiments. To submit microarray experiments to GEA, provision of raw and processed data files, and associated IDF and SDRF metadata are necessary. Raw data of sequencing experiments are stored in DRA and not in GEA.

The GEA accepts functional genomics experiments in a structured and standardized MAGE-TAB format adopted by ArrayExpress (10). A submission to the GEA consists of four parts: raw and processed data, as well as associated metadata in Investigation Description Format (IDF) and in Sample and Data Relationship Format (SDRF) (Figure 2). IDF metadata provides an overview of the experiment, including experimental design, protocols, publication information, and submitter details. SDRF metadata provides sample characteristics and the relation between samples, microarray or sequencing platforms, and raw and processed data files.

The submission workflow of microarray and sequencing experiment involves three and four steps, respectively (Figure 2). The microarray submission consists of (i) preregistration of project information to BioProject, (ii) preregistration of sample information to BioSample and (iii) provision of raw and processed data to the GEA (Figure 2). Major commercial array designs at ArrayExpress can be referenced by citing their array design accession numbers. For a novel array design, the submitter needs to register its Array Design Format file to the GEA.

The sequencing submission consists of four steps. (i) preregistration of project information to BioProject, (ii) preregistration of sample information to BioSample, (iii) preregistration of raw data to the DRA and (iv) provision of processed data to the GEA (Figure 2). The submitter may complete the first three steps through a single DRA submission. Raw sequencing data are stored in the DRA and not in the GEA. This submission process is different from that of the GEO (or ArrayExpress), where the database submission pipeline brokers raw sequencing data of the GEO (or ArrayExpress) into NCBI (or EBI) SRA, respectively.

In the GEA submission portal, the submitter creates IDF and SDRF metadata files associated with raw and processed data. In the IDF tab, the submitter provides an overview of the experiment: title, description, type and design; and protocols, array design (microarray), and publications. In the SDRF tab, the submitter verifies a SDRF template generated from the selected BioSample records, DRA submission (sequencing) or array design (microarray), and the IDF. The submitter is also required to include information describing the material and data files obtained from the samples, as well as the experimental variables under investigation. Lastly, in the ‘Overview’ tab, the submitter confirms the created IDF and SDRF metadata files. Then the system validates the submitted IDF and SDRF files and sends error and warning messages according to validation rules (https://www.ddbj.nig.ac.jp/gea/validation-e.html).

After the metadata and data files are reviewed, the GEA issues unique accession numbers to the experiments and array designs in ‘E-GEAD-*n*’ and ‘A-GEAD-*n*’ formats, respectively (*n* being a number). Because the GEA and ArrayExpress share the accession namespace, the GEA numbers are searchable at ArrayExpress as well. The registered data may be kept private for a limited time, typically during the peer-review process of the relevant publication. The submitter can generate an access token for private GEA data in the submission portal for reviewers. The data would then become public either when the accession number associated with the data is published or at the release date designated by the submitter, whichever comes first. Public GEA experiments and array designs are accessible at its FTP (ftp://ftp.ddbj.nig.ac.jp/ddbj_database/gea) and will be indexed by ArrayExpress. In collaboration with the Database Center for Life Science (DBCLS), GEA metadata will become searchable at the DBCLS All Of gene Expression (AOE, http://aoe.dbcls.jp), an interface where public functional genomics data can be searched and viewed. The DDBJ Center also mirrors ArrayExpress data at its FTP site (ftp://ftp.ddbj.nig.ac.jp/mirror_database/arrayexpress).

The DDBJ Center archived 260 functional genomics experiments at the Center for Information Biology gene EXpression database (CIBEX) (17), which operated from 2003 to 2012. The CIBEX data, with their original CIBEX accession numbers, will be available at the GEA.

## DDBJ SYSTEM UPDATE

### Submission services of biological data

As sequencing technologies are changing and submissions are increasing, our submission processes are also transitioning from manual curation to automatic validation. As part of such efforts, we have implemented the DDBJ BioSample validator and it validates submitted BioSample records according to validation rules, autocorrect formats, and sends error messages and warnings (https://www.ddbj.nig.ac.jp/biosample/validation-e.html). The submitter can correct errors and improve content according to the validation messages displayed in the submission portal. The BioSample submission system automatically assigns accession numbers to submitted records without errors. The BioSample curators have shifted from manual, record-by-record curation to validation rule improvement.

### Retrieval and analysis services of biological data

The DDBJ Center provides web interfaces of the getentry data retrieval system by accession numbers and the ARSA keyword search system for traditional DDBJ flat files (18), Web BLAST (19), ClustalW (20), VecScreen (http://ddbj.nig.ac.jp/vecscreen) and TXSearch (http://ddbj.nig.ac.jp/tx_search) services. For programmatic access to the data, the DDBJ Center also provides the Web API for Bioinformatics (WABI) (21), which includes BLAST, VecScreen, ClustalW, MAFFT (22), getentry and ARSA services. We also have the DDBJ Read Annotation Pipeline (https://p.ddbj.nig.ac.jp), which is the web service for analyzing high-throughput DNA sequencing reads (23).

We provide download services for public data in our archival databases at ftp://ftp.ddbj.nig.ac.jp/ddbj_database/ and those in other mirrored databases at ftp://ftp.ddbj.nig.ac.jp/mirror_database/.

### The NIG supercomputer

The DDBJ Center also operates the NIG supercomputer, a commodity-based computer cluster designed for storing and analyzing large-scale sequencing data. The NIG supercomputer is composed of calculation nodes for general-purpose (554 thin nodes at 64 GB memory each) and memory-intensive tasks, including *de novo* sequence assembly (10 medium nodes, each with 2 TB of memory and one fat node with 10 TB of memory). The calculation nodes are interconnected with InfiniBand, and the total peak performance of its CPUs is 372 Tflops. To support massive I/O in the big-data analysis, the NIG supercomputer is equipped with 10.6 PB of the Lustre parallel distributed file system (http://lustre.org). Large-scale DRA and JGA data are stored in 30 PB of the hierarchical storage system (the IBM Elastic Storage Server). The NIG supercomputer is also installed with major biological datasets and popular bioinformatics tools. Users can transfer large data between their local computers and the NIG supercomputer by using the high-speed-transfer software IBM Aspera (https://asperasoft.com/).

The ever-increasing volume of personal sequencing data makes it difficult for researchers to prepare their own secure computer resources with sufficient storage and computing power and to transfer large data online from public databases including the JGA. To solve these issues, the DDBJ Center has provided a secured NIG supercomputer environment for analyzing personal sequencing data, which is composed of 32 thin nodes and 440 TB of storage and logically separates each user's computing environment (Figure 1). JGA users are allowed to analyze approved JGA datasets in the secured NIG supercomputer as an ‘off-premise server’ in addition to their own servers. Because the secured NIG supercomputer is connected with the JGA server through a high-speed network, users can smoothly download JGA datasets and analyze their own personal genomic data in the same environment. We charge users of the secured supercomputer to share operation and security costs with them (https://sc.ddbj.nig.ac.jp/ja/application/individual-genome-analysis-system, Japanese text only).

## FUTURE DIRECTION

To process the growing number of data submissions and access requests to the NBDC and the JGA, the DDBJ Center and the NBDC are developing an integrated system which will seamlessly connect the NBDC request application processes and the JGA data submission/download processes by using the same user account system.

To manage the increasing volume of sequencing submissions despite limited resources and budget, we are shifting from manual, record-by-record curation to a more automatic validation-based processing system. We are developing validators for BioProject and the DRA and will implement them to the submission systems to automate submission processes. We are also working to automate bacterial genome submissions by using the DDBJ Fast Annotation and Submission Tool (DFAST, https://dfast.nig.ac.jp), which is a bacterial genome annotation pipeline producing submission-ready DDBJ annotation files (24).
